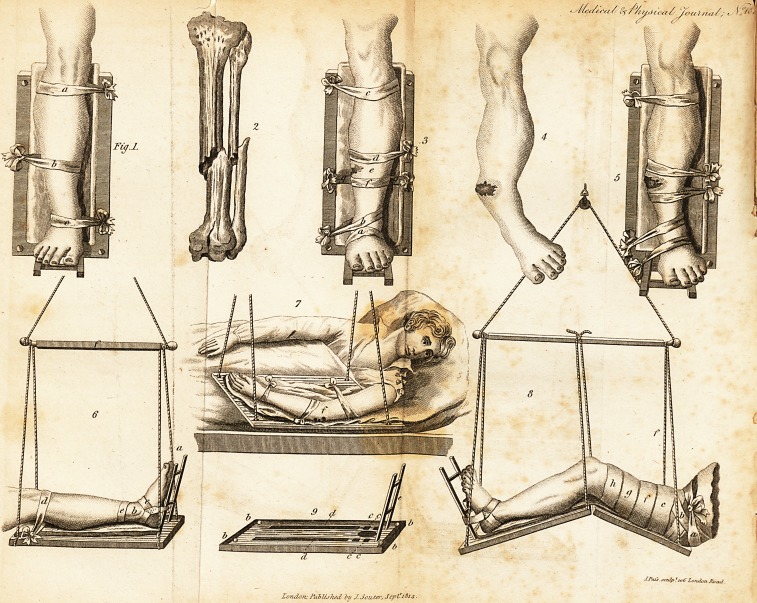# Description of a New Mode of Treating Fractures of the Extremities, from the German of Mr. Sauter

**Published:** 1814-09

**Authors:** 


THE
### Medical and Physical Journal.

3 OF VOL. XXXII.] SEPTEMBER, 1814. [NO. 187.

"For many fortunate discoveries in medicine, and for the detection of numerous errors, the world is indebted to the rapid circulation of Monthly Journals; and there never existed any work to which the faculty in EUROPE and AMERICA were under deeper obligations than to the Medical and Physical Journal of London, now forming a long, but an invaluable series."—RUSH.

### *For the Medical and Physical Journal.*

### *Description of a new Mode of treating Fractures of the Extremities, from the German of Mr. Sauter; by* Mr. WANT.

THE apparatus consists in a board suspended from the bed by cords fixed to the four corners. The fractured limb rests upon this. The leg is fixed to it in three places: first at the knee, then to a frame at the foot, which we shall denominate the foot-board, and the bones are kept in contact by a band at the fractured part, which is fastened to one side of the board. The limb is uncovered and *unincumbered* during the cure, and, once reduced, the apparatus never requires to be altered; the patient may sit and raise himself without in the slightest degree deranging the fracture, which can be daily examined without removing the bandage. In compound fractures which require daily dressing, the importance of this method is inconceivably great. The case which led Mr. Sauter to conceive this plan, was an oblique compound fracture, three inches above the malleolus internus. The superior fragment was completely denuded, and projected an inch from the wound. At every movement of the leg the blood jutted out; the foot was drawn outwards and upwards; the fibula was separated from the tibia; the interosseous ligament was ruptured, and the soft parts were much injured. The fracture was dressed five times in the usual manner; but, notwithstanding the splints and compresses applied in various ways, at every dressing the upper extremity of the tibia was found still projecting from the wound. The limb was erisipelatous, plyctenæ had formed, and the convulsive movements were occasionally so strong that tetanus was apprehended. From the moment this machine was applied, the fractured bones were kept in close contact, and the patient recovered in six weeks.

The machine varies according to the limb fractured, but its mechanism is always the same; that which belongs to fractures of the leg is the most simple, and serves as the basis of the others. It consists in a board of soft wood, 4 feet long by 10 inches wide. (See Fig. 9.) At each angle a hole (*b*) is made for ropes, by which it is to be suspended. At one end two mortises (*c c*), 4 inches distant from each other, to receive the tenons of a frame for supporting the foot. These mortises are in a slightly oblique direction, to give an inclination of about 105 degrees to the foot-board, and several of them may be made at small distances from each other, for the convenience of adapting the machine to limbs of different lengths. An oval hole is made anterior to the foot-board for the heel to rest in.* The frame for supporting the foot is made of two upright pieces of firm wood, 11 inches long and three-quarters of an inch thick; these are connected together by two cross bars. The whole is suspended from the ceiling of the bed by means of two cords, each about 6 feet in length. The two extremities of one of them are passed into the holes (*b b*) of the upper end of the board, Fig. 9. The same is done with the other cord at the other extremity; each cord is then fastened at the middle to the end of a stick of the same length as the board, and from the ends of this stick other cords are passed, by which it is suspended to the ceiling, as may be seen in the Plate.

The bed on which the patient is placed, should be so arranged as to have *nothing beneath* and at the sides of the board which can impede its movement; and the fractured limb should be on a level with the body. In hospitals one may be kept for the purpose. It is merely necessary that the fourth of a mattrass should be removed on the side of the fractured extremity, and of course the lower portion. Where these are not at hand in private families, it will not be difficult to contrive the making of the bed so as to answer the same intention.

It is scarcely necessary to go into minute detail respecting the manner of preparing the bandages, or the pads which must necessarily be placed on the board upon which the fractured limb is to be confined. It will be recollected, that the bandages must be sufficient to keep up a *permanent* extension; and the pads are intended to afford a bed sufficiently easy on which the leg may rest during the progress of the

* If the tenons of the frame were made of some firm material, as steel, they might be reduced considerably in diameter, by which means the distance between the mortises could be much lessened, and less chance given of shortening of the limb. There seems to be no great necessity for the hole intended for the heel to rest in, as the arrangement of the padding may supply its place.

ture, and which must be so arranged as to fill up the inequalities of the under surface of the limb.

The application of the machine will be obvious from the accompanying Plate. The knee should first be firmly fixed to the upper extremity of the board, the foot should be bound to the frame, and the bands of direction must be loosely applied. When the foot is fixed, the extension is to be made when the limb is brought to its original length, the tenons of the frame are to be fixed into the mortises, the limb will then be kept in a state of permanent extension without the possibility of shortening; the bands of direction must then be applied according to the circumstances of the case.

EXPLANATION OF THE PLATES.

Fig. 1.—This figure gives the most simple idea of the bands of direction. The author was called to a fracture twenty days after it had happened. The muscular action had drawn the foot outwards, leaving the limb concave outwardly and convex on the opposite side; by means of the apparatus, and the middle band of direction (*b*), the limb was made perfectly straight.

Fig. 2.—An oblique fracture of the bones of the leg, the superior fragment of which had pierced the integuments.

Fig. 3.—The same fracture with the apparatus applied.

*a*, The band of extension.

*b*, Assists the foregoing, and draws the lower fragment inwards.

*c*, The superior band of direction.

*d*, The band of direction applied near the fracture, which acts in a direction opposite to the two former.

*e*, The wound.

*f*, Another band of direction, to confine the lower fragment which jutted forwards.

Fig. 4.—The leg of a young man after being ten weeks broken, under the care of an unskilful surgeon. The limb remained curved, as is represented in the Plate. At first it was a simple fracture, but from the mismanagement of the surgeon, a considerable ulceration took place, with caries of the bone. It is not necessary to relate the whole particulars of the case, but it shews in a striking manner the utility of our author's practice, where splints could not have been applied on account of the wound and exfoliating bone.

Fig. 5.—The manner of applying the bandages to the foregoing case.

Fig. 6.—The machine applied to the leg, with the bandages for extension and direction.

*a*, The frame or foot-board.

*b*, The wound caused by the projection of the fragment.

*c*, The band of extension, which in this case is attached only to the internal part of the frame.

*d*, The superior band of direction, which should operate on the same side as the band of extension.

*e*, The middle band, which ought to act in a direction contrary to the two former; to draw the upper portion of the fractured bone against the lower, and keep them in contact.

*f*, The stick to which the cords are fastened.

Fig. 7.—The machine applied to a compound fracture of the arm.

*f*, Two wounds near the joint, from which a fragment of carious bone was removed.

Fig. 8.—The application of the machine to the thigh.

Fig. 9.—The board on which the limb is placed.

*b*, The angles where the suspending cords are fixed.

*c*, Mortises, to be increased in number according to circumstances.

*d*, Grooves for the bands of direction.

*e*, Foot-board or frame.

---

*For the Medical and Physical Journal.*

## Figures and Tables

**Fig.1. 2 3 4 5 6 7 8 9 f1:**